# High-Throughput Immunological Analysis of Dictamni Cortex: Implication in the Quality Control of Herbal Medicine

**DOI:** 10.3390/molecules24162880

**Published:** 2019-08-08

**Authors:** Miranda Sin-Man Tsang, Pang-Chui Shaw, Ida Miu-Ting Chu, Ling Cheng, Eric Chun-Wai Wong, David Tai-Wai Lau, Christopher Wai-Kei Lam, Chun-Kwok Wong

**Affiliations:** 1 Institute of Chinese Medicine and State Key Laboratory of Research on Bioactivities and Clinical Applications of Medicinal Plants, The Chinese University of Hong Kong, Hong Kong; 2 Department of Chemical Pathology, The Chinese University of Hong Kong, Prince of Wales Hospital, Hong Kong; 3 School of Life Sciences, The Chinese University of Hong Kong, Hong Kong; 4 Li Dak Sum Yip Yio Chin R & D Centre for Chinese Medicine, The Chinese University of Hong Kong, Hong Kong; 5 State Key Laboratory of Quality Research in Chinese Medicines, Macau Institute for Applied Research in Medicine and Health, Macau University of Science and Technology, Taipa, China

**Keywords:** cytokines, herbal medicine, immune profiles, quality control

## Abstract

Quality inconsistency of herbal medicine is an obstacle that limits the extensive use and study of traditional Chinese medicine. Differences in environmental conditions and processing methods of herbal medicine often result in varying clinical outcomes in patients. Standard chemical markers used for the quality control (QC) of herbal medicine are usually the most abundant and characteristic components, which may not be therapeutically relevant or cannot comprehensively reflect the biological quality of the herbs. In view of this, a novel QC method for better assessment of herbal medicine has been developed via bioactivities analysis. Immunological activities of Dictamni Cortex, a typical herbal medicine for the treatment of various inflammatory diseases, from different geographical locations in China, were evaluated. Upon in vitro treatment of their water and ethanol extracts, distinct patterns of inflammatory cytokines including tumor necrosis factor (TNF)-α, interleukin (IL)-10, IL-6, IL-12p70, IL-1β, and chemokine CXCL8 were released from the lipopolysaccharides- and/or phytohaemagglutinin-stimulated human peripheral blood mononuclear cells (PBMC). Thus, in addition to the commonly used morphological, chemical, or DNA markers, the novel high-throughput profiling of inflammatory cytokines and chemokines of PBMC upon treatment with herbal extracts could be an important reference to help for the quality control of herbal medicine in the future.

## 1. Introduction

Traditional Chinese medicine (TCM) has been used for thousands of years in clinical practice in China. They are gaining acceptance worldwide because they are readily accessible from the natural environment and affordable. Large-scale cultivation of herbal medicine has also been widely adopted in China for meeting the increasing demands for TCM [1]. However, the uncontrolled quality of TCM has caused concerns over their therapeutic reliability and safety. According to the Shen Nong’s Herbal Classic and the famous Chinese medicine practitioner Li Shizhen, environmental factors can seriously influence the nature and properties of herbal medicine [2]. The term “Daodi medicinal material” describes the authenticity and quality of herbal medicine produced from specific geographical regions of China. They were selected based on their specific cultivation location and condition, strict harvesting and processing methods, traditional reputation for their supreme quality, and therapeutic efficacy as proven by long-term clinical trials [2]. Superior herbal medicine can be produced from the Northern (e.g., Hebei and Inner Mongolia), Midland (e.g., Henan and Anhui), and Southern (e.g., Guangxi, Guangdong) parts of China.

Intra-species variations, changes in ecological and environmental conditions (e.g., pesticides and insecticides applied, and soil nutrient and heavy metal contents), seasonal harvesting and storage conditions, and the processing and extraction methods used, are all prone to batch-wise variations in the quality of herbal medicine. It has been reported that nutritional conditions of the soil could vary remarkably across regions within the same province in China [3]. Harvesting time, collection sites, as well as processing methods affect the chemical constituents of herbal medicine [4]. Influences on the bioactive compounds/ingredients or genetic makeup will potentially alter the physical appearances, pharmacological activities, as well as the therapeutic efficacy of the herbs of the same species [5,6,7,8]. Quality inconsistency of herbal medicine also results in reproducibility problems in clinical and experimental research, thereby hampering their usage. 

Currently, the authentication and quality control (QC) of herbal medicine mainly rely on morphological examination, DNA fingerprinting, as well as the identification and quantification of one or two chemical markers in the herbs [9]. Although increasing studies have been conducted to elucidate the bioactivities of these chemical markers; only a minute concentration of these so-called “bioactive” compounds can usually be found in the whole herbal remedy [10]. Therefore, those chemical/molecular markers may not comprehensively reflect the bioactivity and therapeutic efficacy of the herbal medicine [4]. Changes of other unmeasured bioactive components might be neglected in the conventional QC method [9]. 

In this regard, our current study aims at elucidating the possibility of using immunological markers as supplementary parameters for the QC of herbal medicine. Four samples of Dictamni Cortex (Baixianpi) produced from different geographical locations of China (Table 1), were used to illustrate the potential application of this QC method. Dictamni Cortex is the dried root bark of *Dicramnus dasycarpus Turcz*. It possess immunomodulatory effects, and has been traditionally and widely used for treating various inflammatory diseases including jaundice, rheumatism, and cough and skin inflammation including eczema and rubella [11]. The water and ethanol extracts of Dictamni Cortex demonstrated the inhibition in delayed-typed hypersensitivity and an improvement in liver functions in mice [12,13,14]. Anti-allergic and anti-inflammatory effects were also observed upon treatment with Dictamni Cortex extracts [15,16,17]. Ultra-performance liquid chromatography (UPLC) analysis revealed that Dictamni Cortex mainly contains dictamnine and six limonoid derivatives, including fraxinellone, obacunone, limonin, fraxinellonone, rutevin, and dictamdiol [18]. Although numerous studies have reported the anti-inflammatory and anti-microbial functions of chemical constituents in Dictamni Cortex [19,20], only two of the active compounds, obacunone and fraxinellone, are included as the QC markers in the current edition of Chinese Pharmacopoeia [11]. In an attempt to elevate the potential application of the immunological activities for the QC of herbal medicine, inflammatory cytokines released by the macrophage and B lymphocyte mitogen lipopolysaccharides (LPS)- and/or T lymphocyte mitogen phytohemagglutinin (PHA)-stimulated human peripheral blood mononuclear cells (PBMC) upon in vitro incubation with the herbal extract of Dictamni Cortex samples from different places of origins were measured using high-throughput multiplex bead-based assay. The immunological activities of each herbal extract were examined, using PBMC purified from nine human individuals. More importantly, the difference in cytokine profiles between herbal extracts were compared.

## 2. Results

### 2.1. Macroscopic Features of Dictamni Cortex Samples

**Figure 1:**
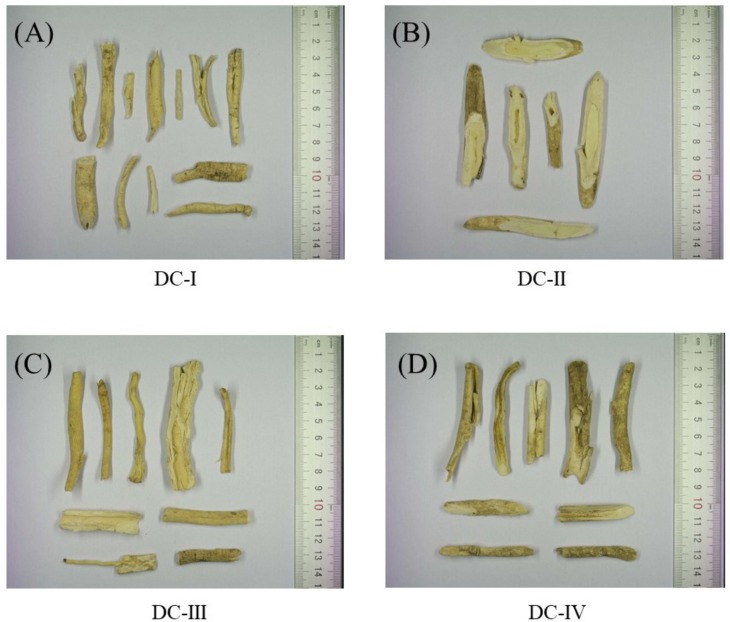
Photographs of the four Dictamni Cortex crude drugs. (**A**) Sample DC-I; (**B**) sample DC-II; (**C**) sample DC-III, and (**D**) sample DC-IV were purchased from the local market. Their places of origin were Inner Mongolia (Region 1), Hebei, Inner Mongolia (Region 2), and Anhui, respectively. DC: Dictamni Cortex.

The places of cultivation origins and endotoxin levels of the four raw samples of Dictamni Cortex [DC-I, DC-II, DC-III, and DC-IV] purchased from the local market are listed in Table 1. The heavy metal and toxic element content, as well as the residual pesticides in the four samples, are listed in Table 2. All samples were authenticated based on their macroscopic features as they all possess a yellow cork, layered cross-sections, and a wide phloem (Figure 1). DC-I, DC-III, and DC-IV were mostly semi-quilled with edges curving inwards; however, DC-II was thicker in diameter with a cylindrical shape. The outer surface of DC-I, DC-II, and DC-III were grayish yellow, while DC-IV had a darker coating.

### 2.2. TLC Profiles of Dictamni Cortex Samples

**Figure 2:**
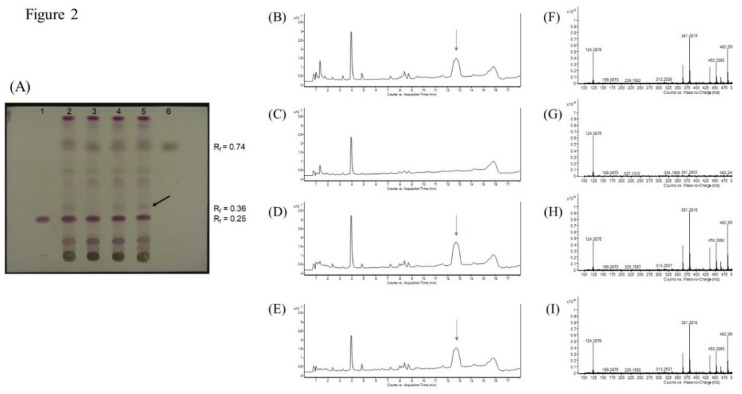
Thin-layer chromatography (TLC) of Dictamni Cortex crude drug samples and liquid chromatography–mass spectrometry (LC/MS) of distinct compounds isolated from Dictamni Cortex crude drug samples. (**A**) TLC documented under visible light. 1, Standard obacunone; 2, Sample DC-I; 3, Sample DC-II; 4, Sample DC-III; 5, Sample DC-IV; and 6, Standard fraxinellone. Refraction factor (Rf) of obacunone and fraxinellone were 0.25 and 0.74, respectively. A representative distinct greyish purple band at R_f_ = 0.36 is denoted by black arrow. (**B**–**E**) Total ion chromatogram (TIC) of Dictamni Cortex crude drug samples DC-I, II, III, and IV, respectively. The characteristic peaks observed in DC-I, DC-III, and DC-IV (retention time = 12.7 min) was denoted by blue arrows. (**F**–**I**) Mass spectrum of the characteristic peak or its equivalent region (retention time = 12.334–13.068 min) of DC-I, DC-II, DC-III, and DC-IV observed in Figure 2B–E. The places of origin of the Dictamni Cortex samples DC-I, II, III, and IV were Inner Mongolia (Region 1), Hebei, Inner Mongolia (Region 2), and Anhui, respectively.

The crude Dictamni Cortex samples were also authenticated based on their chemical profiles using thin-layer chromatography (TLC). All samples of Dictamni Cortex showed a purple band at R_f_ = 0.25 and a grey band at R_f_ = 0.74 that corresponded to the bands observed in the standards obacunone and fraxinellone, respectively (Figure 2A). However, a distinct greyish purple band (R_f_ = 0.36) was exclusively observed in DC-I, DC-III, and DC-IV (lane 2, 4, and 5, respectively). The compounds at R_f_ = 0.36 were isolated, and a characteristic peak with retention time at 12.7 min was observed only in DC-I, DC-III, and DC-IV (Figure 2B,D,E). The mass spectrum of the characteristic peak was also characterized (Figure 2F,H,I). However, the elemental composition and structure of the compounds that specifically present in DC-I, DC-III, and DC-IV could not be further identified based on the mass spectral database available. Nevertheless, the TLC profile of all four crude drug samples were very similar and presented identical bands with chromatographic characteristics that resembled the standards obacunone and fraxinellone as specified in The Chinese Pharmacopoeia [11].

### 2.3. Quantification of Chemical Standards in Dictamni Cortex Samples

**Figure 3:**
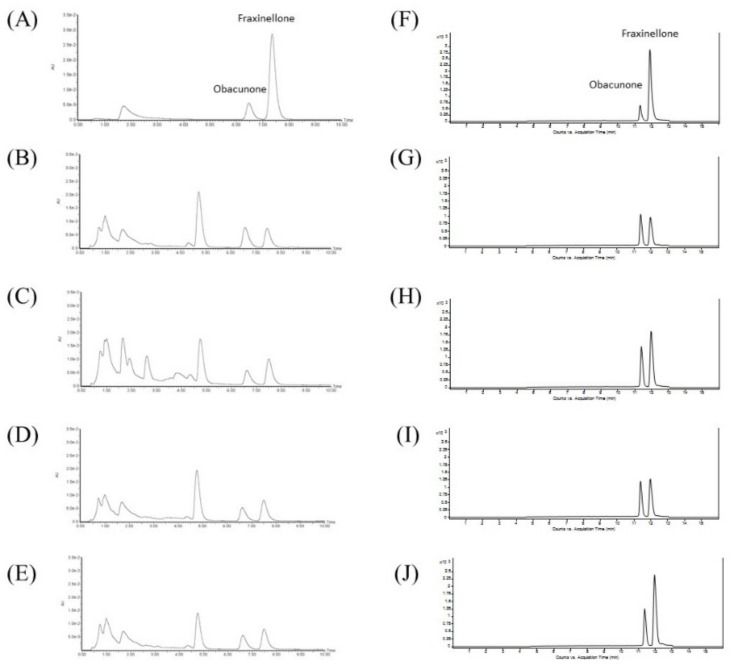
Ultra-performance liquid chromatography (UPLC) and liquid chromatography–mass spectrometry (LC/MS) of Dictamni Cortex crude drugs. (**A**) Standard mixture of obacunone and fraxinellone; (**B**) Sample DC-I; (**C**) Sample DC-II; (**D**) Sample DC-III; and (**E**) Sample DC-IV in UPLC. Retention times of obacunone and fraxinellone were 6.47 and 7.36 min, respectively. (**F**) Standard mixture of obacunone and fraxinellone; (**G**) Sample DC-I; (**H**) Sample DC-II; (**I**) Sample DC-III; and (**J**) Sample DC-IV in LC/MS. Retention times of obacunone and fraxinellone were 11.4 and 11.9 min, respectively. The places of origin of the Dictamni Cortex samples were Inner Mongolia (Region 1), Hebei, Inner Mongolia (Region 2), and Anhui, respectively.

Chemical markers in the crude drugs of Dictamni Cortex were quantified by UPLC and liquid chromatography–mass spectrometry (LC/MS). The peaks of the chemical standards, obacunone and fraxinellone, in the samples were identified by comparing the retention times with those of the standards as listed in The Chinese Pharmacopoeia [11] (Figure 3A). Dictamnine, another abundantly found component in Dictamni Cortex [21], was also identified (Figure 3F). Three replicate injections were performed for each sample, and the mean percentage of each chemical marker was calculated.

All crude samples of Dictamni Cortex revealed the characteristic peaks of the chemical standards obacunone and fraxinellone with retention times at 6.47 and 7.36 min, respectively, in UPLC (Figure 3A–E). About 0.5% obacunone and 0.15% fraxinellone were identified in all samples (Figure 3B–E), which fulfilled the authentication criteria of The Chinese Pharmacopoeia based on the chemical markers. Obacunone (retention time at 11.4 min) and fraxinellone (retention time at 11.9 min) in the crude drugs of Dictamni Cortex were also identified (Figure 3F–J) and quantified by LC/MS (Table 3).

### 2.4. Cell Cytotoxicity and Proliferation of PBMC Upon Treatment with Dictamni Cortex Extracts

All four samples of Dictamni Cortex were extracted separately with water and 95% ethanol. The optimal working concentration of water extracts and ethanol extracts of each samples without inducing significant cytotoxicity or cell proliferation were determined by PI staining and BrdU assays, respectively. 

**Figure 4:**
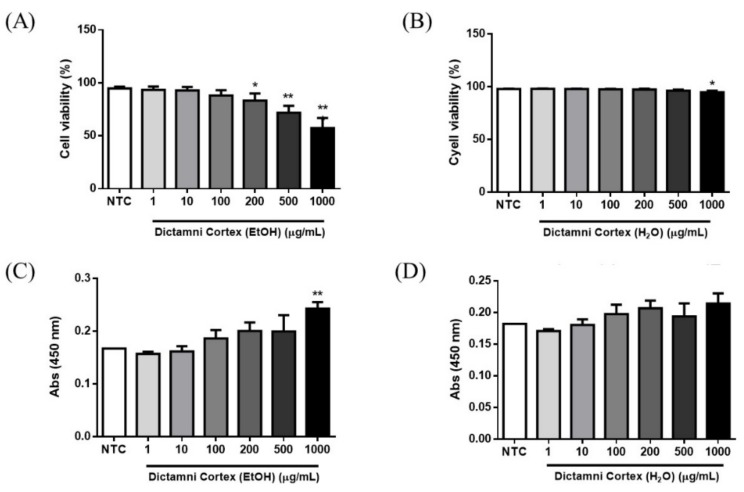
Cytotoxicity and cell proliferation of peripheral blood mononuclear cells (PBMC) upon treatment with Dictamni Cortex extracts. (**A**,**B**) Cell viability of ethanol and water extracts of Dictamni Cortex, respectively. Heat-killed PBMC were used for the gating on the FACSVia flow cytometer. Dead cells were determined by the positive staining with PI. The cell viability (%) was calculated by subtracting 100 with the percentage of PI^+^ cells. Cell viability (%) of all samples was shown in bar chart with mean + S.E.M. (**C**,**D**) Cell proliferation of ethanol and water extracts of Dictamni Cortex, respectively. Cell proliferation was measured by BrdU assays. Absorbance results at 450 nm are shown in bar charts with mean + S.E.M. * *p* < 0.05, ** *p* < 0.01 when compared with the untreated control in triplicate experiments. NTC, untreated control.

Considerable dose-dependent effects on the cytotoxicity of the ethanol extracts of all Dictamni Cortex samples were observed. However, ethanol extracts (100 µg/mL) and water extracts (500 µg/mL) did not significantly reduce the cell viability of PBMC (Figure 4A,B); while the ethanol extracts (500 µg/mL) and water extracts (1000 µg/mL) did not induce significant cell proliferation of PBMC (all *p* > 0.05, Figure 4C,D).

### 2.5. Cytokine/Chemokine Profiles of Dictamni Cortex Ethanol Extracts

**Figure 5:**
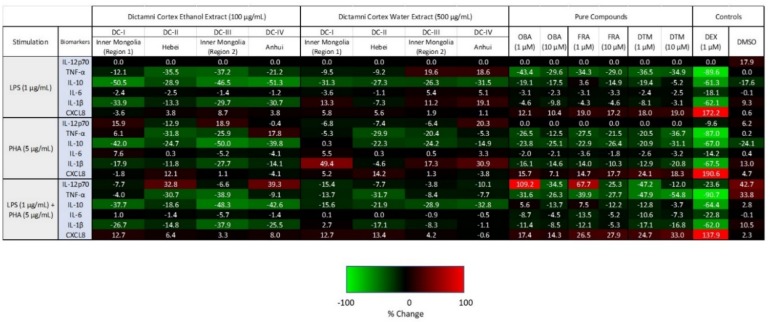
Cytokine/chemokine heat map of PBMC upon treatment with Dictamni Cortex ethanol extract (100 µg/mL), water extract (500 µg/mL), obacunone (OBA) (1, 10 µM), fraxinellone (FRA) (1, 10 µM), dictamnine (DTM) (1, 10 µM), dexamethasone (DEX) (1 µM), and dimethyl sulfoxide (DMSO) (0.1%) with 1 µg/mL LPS and/or 5 µg/mL PHA stimulation for 24 h. Cytokines released by PBMC (1 × 10^5^) were quantified by cytometric bead array assay. The average percentage changes in cytokine/chemokine relative to the untreated controls are shown in heat map with spectral colors. Upregulated cytokines are shown in red, with bright red indicating strongly upregulated cytokines (i.e., upregulated by 100%). Downregulated cytokines are shown in green, with light green indicating cytokines that were strongly downregulated (i.e., downregulated by 100%). Unchanged cytokines are shown in black. DC-I, II, III, and IV were the Dictamni Cortex extracts prepared from the Dictamni Cortex crude drugs from Inner Mongolia (Region 1), Hebei, Inner Mongolia (Region 2), and Anhui, respectively.

Cytokines IL-12p70, IL-1β, IL-10, IL-6, TNF-α, and CXCL8 released from the LPS and/or PHA-stimulated PBMC were quantified by cytometric bead array (CBA) assay using flow cytometry. For better visualization of the changes in the release of these cytokines, the percentage change upon the treatment with herbal extracts were illustrated in heat maps with spectral colors (Figure 5). 

Upon LPS stimulation, cytokine profiles of the four Dictamni Cortex ethanol extracts (100 µg/mL) were generally identical, with a down-regulation in the release of TNF-α, IL-10, and IL-1β while keeping the IL-12p70 and IL-6 unchanged. 

Interestingly, upon PHA stimulation, distinct cytokine patterns were found among the four Dictamni Cortex ethanol extracts. TNF-α was upregulated by DC-I and DC-IV; however, it was down-regulated by DC-II and DC-III. IL-6 was exclusively stimulated by DC-I. On the other hand, DC-II stimulated the release of CXCL8. Strikingly, both DC-I and DC-III, which are samples produced from Inner Mongolia, revealed a similar cytokine profile, with an upregulation in IL-12p70, but a consistent suppression in CXCL8. 

Upon LPS and PHA co-stimulations, the difference in cytokine profiles among the four Dictamni Cortex ethanol extracts became more recognizable, with DC-I and DC-III showing the most similar suppressive effect in the release of screened inflammatory cytokines. 

Taken together, the four ethanol extracts of Dictamni Cortex samples exhibited differential suppressive effect on the release of cytokines from PBMC, especially upon PHA and LPS-PHA co-stimulation.

### 2.6. Cytokine/Chemokine Profiles of Dictamni Cortex Water Extracts

Upon LPS stimulation, certain similarity was observed among the four Dictamni Cortex water extracts (Figure 5). All water extracts showed a downregulation in IL-10, and an unchanged IL-12p70 level. Notably, in terms of the release of TNF-α, IL-6, and CXCL8, the cytokine profiles of DC-I and DC-II were entirely the opposite of DC-III and DC-IV. 

Upon PHA stimulation, very distinct cytokine patterns were observed among the four Dictamni Cortex water extracts. Remarkably, only DC-II showed a reduction in the release of IL-1β but a stimulation in the release of CXCL8. Nevertheless, TNF-α was consistently increased in all the four Dictamni Cortex water extracts.

Upon LPS and PHA co-stimulation, although the cytokine profiles of the four Dictamni Cortex samples were similar, contrasting patterns could still be observed in the release of CXCL8. DC-I, DC-II, and DC-III showed a moderate upregulation in CXCL8; while DC-IV showed an unchanged CXCL8 release. The cytokine profiles among the four water extracts showed a more prominent differentiation upon LPS or PHA stimulation when compared to the co-stimulations. 

The cytokine patterns of the pure compounds obacunone (OBA), fraxinellone (FRA), and dictamnine (DTM) upon LPS and/or PHA stimulation were highly comparable (Figure 5). Upon LPS or PHA stimulation, all pure compounds (1, 10 µM) consistently suppressed the release of TNF-α, IL-10, IL-1β; moderately reduced the IL-6; but stimulated CXCL8. Upon LPS and PHA co-stimulations, the reduction of IL-12p70 and IL-6 level by the OBA, FRA, and DTM became more notable when compared to the stimulation with LPS or PHA alone. Immunosuppressive synthetic corticosteroid Dexamethasone (DEX) (1 µM) served as a positive control. 

## 3. Discussion 

Quality control of herbal medicine is paramount in the development of TCM. Quality inconsistency caused by the variation in cultivation conditions and processing methods not only results in unpredictable therapeutic efficacy and safety problem on patients, but also hinders the evidence-based research of Chinese Medicine. This study, for the very first time, demonstrates that bioactive quality of herbal medicine from different geographical regions with the same morphological and chemical marker features, could vary in vitro, and could be evaluated by immune-mediated cytokine profiling. Dictamni Cortex, a common herbal medicine with anti-inflammatory activity, was used to illustrate the possibility of using high-throughput cytokine profiles for the evaluation of the pharmacological quality of herbal medicine. 

Four samples with the name of “Dictamni Cortex” were purchased by experts from a validated Traditional Chinese Medicine Center in Hong Kong. These samples were produced from two different regions of Inner Mongolia, Hebei and Anhui, which represent the most Northern and Eastern parts of China, respectively. The drastic difference in the place of origins reflects the diverse environmental conditions during the cultivation of these samples. Based on the macroscopic examination, as well as the TLC and UPLC analysis (Figure 1, Figure 2 and Figure 3), all samples of Dictamni Cortex purchased in the current study were authenticated according to the guidelines from the Chinese Pharmacopoeia 2015 [11]. The contents of the pure compounds including obacunone, fraxinellone, and dictamnine of the crude samples were quantified by LC/MS analysis (Table 3). Changes in cytokines released from PBMC, upon LPS and/or PHA stimulations, together with the treatment of the Dictamni Cortex water/ethanol extracts were shown in a heat map for easy visualization and comparison. 

The ethanol extracts upon LPS stimulation, and both the water and ethanol extracts upon LPS and PHA co-stimulations, showed largely similar cytokine patterns, demonstrating a general downregulation of inflammatory cytokines, across the four tested Dictamni Cortex samples (Figure 5). Nevertheless, the current study also revealed that Dictamni Cortex from different places of origin possess distinct anti-inflammatory activities using in vitro cytokine induction assay, even though they have similar macroscopic and chemical properties. The cytokine profile of Dictamni Cortex sample from Hebei [DC-II] was particularly different from those produced from Inner Mongolia and Anhui [DC-I, DC-III, and DC-IV]. The absence of a characteristic band at R_f_ = 0.36 of the TLC plate of DC-II (Figure 2A) may account for the immunological difference in PBMC observed in the current study. Isolation of the characteristic band and LC/MS analysis further elucidated the mass spectra of the distinct compounds that exclusively present in DC-I, DC-III, and DC-IV (Figure 2F–I); however, the identity of this distinct unknown compound(s) and their roles on the biological activity of PBMC is yet to be discovered. 

The ethanol extracts of Dictamni Cortex from the two regions of Inner Mongolia [DC-I and DC-III] also revealed substantially similar cytokine patterns upon LPS and/or PHA stimulation, which is drastically different from the Dictamni Cortex produced from Hebei [DC-II] and Anhui [DC-IV]. However, the pro-inflammatory cytokines DC-I and DC-III were not entirely identical. Upon PHA stimulation, the release of TNF-α and IL-6 from the ethanol extract of DC-I and DC-III were drastically different, despite their proximal places of origin. Immunological differences between DC-I and DC-III were also observed in the release of TNF-α and IL-6 from the water extract upon LPS stimulation; IL-10 from the water extract upon PHA stimulation; and CXCL8 from both their water and ethanol extracts upon LPS and PHA co-stimulations. The discrepancy in cytokine profiles observed among the four Dictamni Cortex samples might be explained by the difference in the chemical composition, potentially caused by the different cultivation and processing methods adopted by manufacturing companies. Our results indicate that LPS and/or PHA-induced TNF-α, IL-6, and CXCL8, the common biologically relevant immunological markers in inflammatory diseases, can be useful markers for the evaluation of the pharmacological quality of Dictamni Cortex and other anti-inflammatory herbal medicine. Cytokine profiles of PBMC upon treatment with obacunone, fraxinellone, and dictamnine, the pure compounds commonly found in Dictamni Cortex, together with the commercially available dexamethasone, can be used as anti-inflammatory controls for the current QC of Dictamni Cortex. 

The cytokine profiles of the Dictamni Cortex extracts generated upon LPS and PHA co-stimulations did not show much similarity as those generated upon LPS or PHA stimulation alone. Since LPS and PHA directly and indirectly stimulate the activation and interaction of both innate and adaptive cells in PBMC, the extracts of the Dictamni Cortex, possibly disrupted the inflammatory cascade, leading to a general down-regulation of cytokine release across the four samples. 

The in vitro screening of cytokine levels released by PBMC upon LPS and/or PHA stimulations is biologically relevant. Human PBMC are crucial circulating immune effector cells including macrophages, monocytes, and T and B lymphocytes in various inflammatory diseases; and can actively produce inflammatory cytokines to trigger innate and adaptive immune responses upon stimulation and pathogen invasion [22]. Therefore, the induction of cytokine release could be affected by the number of PBMC. Water and ethanol extract of Dictamni Cortex at a concentration that insignificantly induced cell cytotoxicity and proliferation on PBMC were therefore determined and used for the quality evaluation of the Dictamni Cortex samples (Figure 4). 

Since PBMC are sensitive to external stimuli via the putative receptors such as pattern recognition receptors, the release of cytokines by PBMC could also be largely affected by the endotoxin/LPS contamination in the herbal extracts. Nevertheless, the current study demonstrated the endotoxin levels in the four Dictamni Cortex samples were generally very low or even undetectable, especially in the ethanol extracts (Table 1). The content of heavy metals, toxic elements, and pesticide residues are the potential contaminants during the cultivation and processing of herbal medicine. They were generally comparable in the four Dictamni Cortex samples (Table 2). Although the levels of Cadmium and Lead showed variation among samples (Table 2), their levels did not exceed the limits as recommended by the World Health Organization (WHO) [23]. The storage conditions of the four Dictamni Cortex samples upon purchase were also controlled; and the samples were well documented in a museum. Therefore, the differences in cytokine profile between Dictamni Cortex samples could indicate the intrinsic biological difference of the herbs but not the environmental factors such as contaminants. 

The cytokines released upon the treatment with Dictamni Cortex samples has been normalized with the level of the cytokines stimulated by the LPS and/or PHA; hence, no blank control (cells only without any treatment) or stimulation control (LPS/PHA treatment only without any herbal extract) of PBMC in response to LPS and/or PHA stimulation were shown in the heat map. The average of percentage changes of cytokine released by PBMC from nine human individuals was used to illustrate the anti-inflammatory activities of Dictamni Cortex in the current study, so the inter-individual variation in PBMC purified from different human donors should be minimized. Moreover, PBMC freshly purified in the current study were cultured in the standardized RPMI medium immediately after sufficient washing steps. Variations in the plasma proteins bound to the PBMC caused by factors such as nutritional and physiological differences of the donors were minimized [24,25]. 

Macrophage and B lymphocyte mitogen LPS and T lymphocyte mitogen PHA are two typical stimulatory agents that have been widely utilized to mimic inflammation in vitro. As the typical pathogen-associated molecular patterns on gram-negative bacterial cell wall [26], LPS can be recognized by the toll-like receptor 4 on the antigen-presenting dendritic cells, macrophages, and monocytes in PBMC. LPS can also act as a mitogen that triggers the proliferation and differentiation of B cells and macrophages [24]. This, in turn, drives the production of pro-inflammatory cytokines that mount inflammation. PHA, on the other hand, is a lectin mitogen and polyclonal activator of T lymphocytes [27,28], that can be used to mimic antigen-induced lymphocytes activation and the subsequent magnified immune response. The LPS and/or PHA stimulations were employed in the current study to elaborate the anti-inflammatory activities of the Dictamni Cortex extracts, making the QC assessment biologically relevant. 

Dictamni Cortex has been used to treat various inflammatory diseases [11]. Six representative inflammatory cytokines, IL-12p70, TNF-α, IL-10, IL-6, IL-1β, and CXCL8, were selected to be the immune profiling markers of Dictamni Cortex because these cytokines can generally reflect an inflammatory status [29]. The levels of TNF-α and IL-6 were particularly reduced in mice with skin inflammation upon Dictamni Cortex treatment [16]. Constituents with different polarities extracted by different extraction methods may lead to distinct cytokine profiles found in the water and ethanol extracts of a Dictamni Cortex sample. Therefore, the immunological effects of the four samples of Dictamni Cortex were comprehensively revealed in both water and ethanol extracts in this study. 

The novel QC method employed in the current study allows high-throughput screening for the biological quality of herbal medicine. Firstly, the high abundance of PBMC purified from each bag of human buffy coat allows the screening of multiple herbal medicine at various concentrations at the same time. Furthermore, the selected cytokines can be released within 12 h upon LPS stimulation in vitro [30], making the test very rapid when compared to in vivo testing. The quantification of the cytokines involves the use of multiplex assays, where six or more immune parameters could be measured simultaneously in a tiny amount of sample using commercially available kits accompanied with automatic washing and detection steps, allowing high-throughput data to be generated. Compared with the conventional manual handling ELISA, which only measure one cytokine per assay, the present multiplex assays can save about 10 times the assay time and human effort, providing better sensitivity and coefficient of variation and a wider dynamic range, and minimizing human errors. Therefore, it is less time-consuming and less labor-intensive. The accurate and high-throughput cytokines measurement used in this study allows the generation of specific immune profiles for the QC of herbal medicine. 

Besides, unlike the physical authentication method, where intact herbal samples and experienced experts are required, the novel method described in this study allows the quality screening of both intact and processed samples. This makes the quality evaluation of herbal medicine more applicable in routine operation. Unlike the chemical profiling, the current assay method does not require organic solvents for classical extraction and chemical analysis, which made the method more convenient and environmentally friendly. Taken together, the current study elucidates that biological markers such as inflammatory cytokine/chemokine patterns generated in in vitro assays can act as a supplementary information for the QC of herbal medicine with potential therapeutic relevance. This can also be translated to the QC of other herbal medicine with different clinical use. Biological assays with a specific set of biological parameters can be screened, and stimulants and cell lines can be designed for evaluation of the biological quality of a certain herbal medicine. With the use of biological markers for the QC of herbal medicine in clinical studies, it is also expected that a more consistent results could be achieved in the future. The limitation of this TCM QC application includes the investment of the sophisticated cytokine/chemokine analyzer, the requirement of well-trained technical staff, and relatively high cost of the assay. However, along with the increasing popularity of this high-throughput cytokine/chemokine assay, the cost of the instrumentation and cytokine assay reagent is expected to be decreased. 

In summary, under LPS and/or PHA in vitro stimulation, different cytokine patterns were observed across the four Dictamni Cortex samples. This indicates that samples produced from different regions of China may possess distinct immunological activities, which may allow the quality assessment of herbal medicines using immune method. With this high-throughput cytokine measurement together with the in vivo model for validation, it is possible that minute differences in the immunomodulatory activity of different grades of herbal medicines could be specifically identified and interpreted. Nevertheless, the bioactive ingredients of the four examined samples accounting for the distinct cytokine response need further evaluation. 

## 4. Materials and Methods

### 4.1. Authentications and Storage of Dictamni Cortex Samples

Four samples of Dictamni Cortex, DC-1, DC-II, DC-III, and DC-IV, produced in Inner Mongolia (Region 1), Hebei, Inner Mongolia (Region 2), and Anhui, respectively, were authenticated based on their macroscopic characteristics and chemical profiles, according to The 2015 Edition of Chinese Pharmacopoeia [11]. The chemical composition of the samples was determined by thin-layer chromatography (TLC) with optimization. In brief, crude drug samples ground in powder form were dissolved in methanol with sonication for 30 min. The filtered solutions were dried and dissolved in fresh methanol. The samples, as well as the standards obacunone (National Institutes for Food and Drug Control, China) and fraxinellone (National Institutes for Food and Drug Control), were spotted on the TLC Silica gel 60 F254 plate (Merck Millipore, Temecula, CA, USA). The plate was then allowed to develop over a path of about 8.5 cm in freshly prepared solvent system, methylbenzene: cyclohexane: ethyl acetate (3:3:3). It was sprayed with diluted vanillin-sulphuric acid (5%, *v/v*), heated at 105 °C, and examined under visible light using the Digital Documentation System DD 50 with the ProViDoc software (Desaga Sarstedt-Gruppe GmbH, Wiesloch, Germany). The retardation factor (Rf) value was calculated. All samples were kept in an air-tight container with desiccators in a controlled laboratory condition, with humidity at 46% and temperature at 25 °C. A voucher specimen of each sample [DC-I, DC-II, DC-III, and DC-IV] was well-documented (#3586, #3587, #3593, #3594, respectively) and deposited in the Chinese Medicine Museum, Institute of Chinese Medicine, The Chinese University of Hong Kong.

### 4.2. Quality Assessment by UPLC

The quality of the four Dictamni Cortex samples were assessed by UPLC, as stated in the requirement of The Chinese Pharmacopoeia [11]. The crude drug samples dissolved in methanol (5 µL) was injected into a Waters ACQUITY UPLC BEH C18 column (1.7 µm, 2.1 × 100 mm) (Waters Corp., Milford, MA, USA) of the Waters ACQUITY UPLC System (Waters Corp.), equipped with Waters ACQUITY UPLC BEH C18 guard column (1.7 µm, 2.1 × 5 mm) (Waters Corp.). The solvent system methanol: water, 50:50 under isocratic condition was employed. The flow rate was 0.3 mL/min; and the detection was performed at UV 236 nm. The mixture of obacunone (National Institutes for Food and Drug Control) (retention time: 6.47 min) and fraxinellone (National Institute for Food and Drug Control) (retention time: 7.36 min) was prepared as the standards and analyzed. Chemical markers were identified by comparing the retention times and the UV absorbance of the unknown peaks to those of the standards. 

### 4.3. Quality Assessment of Dictamni Cortex Samples by LC/MS

The content of obacunone, fraxinellone, and dictamnine in the four Dictamni Cortex crude drug samples were quantified by LC/MS. Briefly, the crude drug samples dissolved in methanol (1 µL) was injected into an Agilent ZORBAX Eclipse Plus C18 column (1.8 µm, 3.0 × 100 mm) (Agilent Technologies, Santa Clara, CA, USA), equipped with Agilent ZORBAX Eclipse Plus C18 (1.8 µm, 3.0 x 10 mm) guard column (Agilent Technologies). Gradient elution with solvent system 0.1% formic acid in water and 0.1% formic acid in methanol was used. The mixture of obacunone (Chengdu Must Bio-Technology Co., Ltd., Chengdu, China) (retention time: 11.4 min) and fraxinellone (Chengdu Must Bio-Technology Co., Ltd.) (retention time: 11.9 min) was prepared as the standard and analyzed. Dictamnine (Chengdu Must Bio-Technology Co., Ltd.) (retention time: 5.7 min) was also used as a standard and analyzed. Chemical markers were identified based on the retention time and the UV absorbance that resemble the standards.

### 4.4. Isolation and Characterization of Distinct Compounds in Dictamni Cortex Samples by TLC and LC/MS

The distinct band (R_f_ = 0.36) identified on the TLC plates of DC-I, DC-III, and DC-IV were isolated and characterized by LC/MS. The colorless band area with R_f_ = 0.36 was marked and excised. The compounds in that band area were dissolved in 95% ethanol, filtered, and dried. The weighted powder was then dissolved in methanol for LC/MS analysis. The LC/MS were performed in gradient elution as previously described, with 0.1% formic acid in water and 0.1% formic acid in methanol as the solvent system for the characterization of compounds in the distinct band.

### 4.5. Water and Ethanol Extraction of Dictamni Cortex Samples

Each Dictamni Cortex sample (50 g), soaked in 1 L distilled water, was refluxed in 1 L distilled water at 100 °C for an hour, twice. The water extract was filtered and lyophilized into powder. Similarly, 100 g of Dictamni Cortex sample was extracted by refluxing in 1 L 95% ethanol, twice. The ethanol extract was filtered and condensed using a rotary evaporator connected with a Low Temperature Circulator CoolAce (EYELA USA, Bohemia, NY, USA). All extracted samples were kept in air-tight containers with desiccators under a controlled laboratory condition. Endotoxin levels of the water and ethanol extracts of Dictamni Cortex were quantified by the PyroGene Recombinant Factor C Endotoxin Detection Assay (LONZA, Basel Switzerland). The content of heavy metals, toxic element, and pesticide residues were measured by ALS Technichem (HK) Pty Ltd., Hong Kong.

### 4.6. Purification of Peripheral Blood Mononuclear Cells (PBMC) from Buffy Coat and Cell Culture

Human buffy coat from Hong Kong Red Cross Blood Transfusion Service was diluted in 1:1 with phosphate-buffered saline (PBS). The diluted buffy coat was centrifuged with density gradient media Ficoll-Paque PLUS (GE Healthcare, Little Chalfont, United Kingdom) in 1:1 ratio at 1800 rpm for 25 min at 18 °C. The fraction in the plasma-Ficoll interface was collected. The red blood cells in the fraction were lysed and the fraction was washed twice with cold PBS supplemented with 2% fetal bovine serum (FBS) (Gibco, Carlsbad, CA, USA). The cells (1 × 10^6^/mL) were then counted and cultured in RPMI 1640 medium (Gibco) containing 10% FBS. LPS (1 μg/mL) (Sigma-Aldrich, St. Louis, MO, USA) and/or PHA (5 μg/mL) (Sigma-Aldrich) were added as the stimulants. The PBMC were incubated with each extract (1, 10, 100, 200, 500, and 1000 μg/mL) at 37 °C in a humidified incubator supplied with 5% carbon dioxide and 95% air for 24 h. Cytokine/chemokine changes upon treatment with dexamethasone (DEX) (1 µM), Dimethyl sulfoxide (DMSO) (0.1%) (Sigma-Aldrich), as well as obacunone (Chengdu Must Bio-Technology Co., Ltd.), fraxinellone (Chengdu Must Bio-Technology Co., Ltd.), or dictamnine (Chengdu Must Bio-Technology Co., Ltd.) (1, 10 µM) were used as controls.

### 4.7. Cell Cytotoxicity Assay

Cell cytotoxicity of PBMC upon treatment with herbal extracts [DC-I to DC-IV] was determined by FITC Annexin V Apoptosis Detection kit (BD Biosciences, San Jose, CA, USA). Each herbal extract in various concentrations (0, 1, 10, 100, 200, 500, and 1000 μg/mL) was cultured with PBMC (1 x 10^5^/mL) for 24 h. Upon treatment, PBMC were harvested, washed with ice-cold PBS, and resuspended in binding buffer at 1 x 10^6^/mL. PBMC were stained with propidium iodide solution and quantified by BD FACSVia flow cytometer (BD Biosciences). The heat-killed PBMC at 60 °C for 30 min were used as positive control.

### 4.8. Cell Proliferation Assay

Upon treatments of Dictamni Cortex extracts [DC-I to DC-IV], cell proliferation of PBMC was quantified by BrdU Cell Proliferation Assay Kit (BioVision, Milpitas, CA, USA) according to the manufacturer’s protocol with optimization. Briefly, each herbal extract in various concentrations (0, 1, 10, 100, 200, 500, and 1000 μg/mL) was cultured with PBMC (1 × 10^5^/mL) for 22.5 h. The cell culture was incubated with BrdU solution at 37 °C for 1.5 h. The cells were then incubated with fixing/denaturing solution at room temperature for 30 min, followed by incubation with BrdU Detection antibody for 1 h. The cells were washed, and anti-mouse HRP-conjugated antibody was incorporated. Upon washing, TMB substrate was added. Color development was determined at 450 nm with the VICTOR^3^ Multilabel Plate Reader (PerkinElmer, Waltham, MA, USA).

### 4.9. Quantitative Analysis of Cytokines and Chemokines by Cytometric Bead Array (CBA)

Upon 24-h treatment with either ethanol or water extracts of Dictamni Cortex [DC-I to DC-IV], PBMC were centrifuged at 300× *g* for 10 min. The supernatant of the cell culture was collected. Concentrations of inflammatory cytokine IL-12p70, TNF-α, IL-10, IL-6, IL-1β, and CXCL8 in the supernatant were quantified using the CBA with flow cytometry (BD FACSVia flow cytometer). The percentage change of the release of cytokines and chemokines upon herbal extract treatments as well as LPS, PHA, and LPS plus PHA stimulations were calculated by [(Cytokine _Stimulation + DC extracts_ − Cytokine _stimulation only_)/Cytokine _stimulation only_] × 100%. The average in percentage change of at least three independent experiments was then shown in heat maps created by Microsoft Excel.

### 4.10. Data Analysis

The chromatographic characteristics of the bands, including the color and retention factor (R_f_), of the samples were compared with the standards specified by The Chinese Pharmacopoeia [11]. Mass spectrum of the isolated compound(s) from the distinct bands of the samples was analyzed by Agilent Technologies MassHunter PCDL Manger (Agilent Technologies). All in vitro experiments were performed at least three times. Results were analyzed using Student’s t-tests for comparisons using GraphPad PRISM software version 6.01. A significant level with *p* < 0.05 was considered significantly different.

## 5. Conclusions

We have observed that the water and ethanol extracts of four Dictamni Cortex medicine obtained from four regions of China induced different patterns of cytokines and chemokine stimulation and suppression, when incubated with human PBMC. These immunological parameters of important biological relevance can be used as a supplementary quality control method for strengthening the currently used TLC, UPLC, mass spectrometry, and DNA analysis for the quality assessment of Dictamni Cortex. Such cytokine and chemokine patterns can be determined using high-throughput methodology and instrumentation. Further development, optimization, and establishment of the novel immunological method will facilitate the standardization and modernization, as well as safe and effective application of TCM in future.

## Figures and Tables

**Table 1:** Samples and their origin of Dictamni Cortex and the endotoxin levels of their water and ethanol extracts determined by the Pyrogene Recombinant Factor C Endotoxin Detection Assay.

Herbal Medicine	Samples	Places of Origin	Endotoxin Level (EU/μg)	
			Water Extract	Ethanol Extract
Dictamni Cortex	DC-I	Inner Mongolia (Region 1)	0.01	UD
	DC-II	Hebei	UD	UD
	DC-III	Inner Mongolia (Region 2)	0.57	UD
	DC-IV	Anhui	4.73	UD

UD: undetectable.

**Table 2:** Heavy metals, toxic element, and pesticide residues content in the sample of Dictamni Cortex.

	DC-I (mg/kg)	DC-II (mg/kg)	DC-III (mg/kg)	DC-IV (mg/kg)
Heavy Metals and Toxic Element				
Arsenic	<0.5	<0.5	<0.5	<0.5
Cadmium	0.02	<0.02	<0.02	<0.02
Lead	0.16	0.17	0.12	0.28
Mercury	<0.05	<0.05	<0.05	<0.05
Pesticide Residues				
Aldrin ^A^	<0.05	<0.05	<0.05	<0.05
Chlordane ^B^	<0.05	<0.05	<0.05	<0.05
DDT ^C^	<1.0	<1.0	<1.0	<1.0
Endrin	<0.05	<0.05	<0.05	<0.05
Heptachlor ^D^	<0.05	<0.05	<0.05	<0.05
Hexachlorobenzene	<0.1	<0.1	<0.1	<0.1
Hexachlorocyclohexane ^E^	<0.3	<0.3	<0.3	<0.3
Lindane	<0.6	<0.6	<0.6	<0.6
Quintozene ^F^	<1.0	<1.0	<1.0	<1.0

^A^ As sum of Aldrin and Dieldrin; ^B^ As sum of cis and trans-chlordane and oxychlordane; ^C^ As sum of p,p′DDT, o,p′-DDT,′-DDE, and ′-DDD; ^D^ As sum of heptachlor and heptachlor epoxide; ^E^ As sum of a-, b-, and d-hexachlorocyclohexanes; ^F^ As sum of quintozene, pentachloroaniline, and methylpentachlorophenyl sulphide (ALS Technichem (HK) Pty Ltd.).

**Table 3:** Abundance of obacunone, fraxinellone, and dictamnine in the crude Dictamni Cortex samples determined by UPLC and LC/MS.

		DC-I	DC-II	DC-III	DC-IV
UPLC	Obacunone	0.591%	0.479%	0.506%	0.508%
	Fraxinellone	0.108%	0.164%	0.152%	0.142%
LC/MS	Obacunone	0.372%	0.481%	0.421%	0.438%
	Fraxinellone	0.064%	0.130%	0.086%	0.158%
	Dictamnine	0.027%	0.033%	0.043%	0.029%
